# Case Report: A novel hemizygous missense *PDHA1* variant in a Vietnamese boy with pyruvate dehydrogenase E1-alpha deficiency

**DOI:** 10.3389/fped.2024.1494604

**Published:** 2024-12-10

**Authors:** Thi Thanh Ngan Nguyen, Nguyen Ngoc Khanh, Chi Dung Vu, Ngoc-Lan Nguyen, Van Khanh Tran, Nguyen Thi Kim Lien, Nguyen Van Tung, Nguyen Duc Quan, Nguyen Thanh Hien, Tran Thi Huong Giang, Nguyen Thi Xuan, Nguyen Thien Tao, Tran Van Khoa, Huy Hoang Nguyen

**Affiliations:** ^1^ Institute of Genome Research, Vietnam Academy of Science and Technology (VAST), Hanoi, Vietnam; ^2^ Center of Endocrinology, Metabolism, Genetic/Genomics and Molecular Therapy, Vietnam National Children's Hospital, Hanoi, Vietnam; ^3^ Center for Gene and Protein Research, Hanoi Medical University, Hanoi, Vietnam; ^4^ Department of Biology and Medical Genetics, Vietnam Military Medical University, Hanoi, Vietnam

**Keywords:** *PDHA1*, whole-exome sequencing, missense variant, pyruvate dehydrogenase E1-alpha deficiency, Vietnamese

## Abstract

A pyruvate dehydrogenase complex deficiency causes a reduction in adenosine triphosphate production and energy insufficiency, leading to neurological disorders. An abnormal E1-alpha protein originating from the *PDHA1* gene with pathogenic variants is unable to communicate with E1-beta for the formation of the E1 enzyme, decreasing pyruvate dehydrogenase complex activity. In this study, we report a Vietnamese boy with lethargy, severe metabolic acidosis, increased serum lactate, hyperalaninemia, lactic acidosis, and globus pallidus lesions. Whole-exome sequencing and variant filtering identified a hemizygous missense variant NM000284.4 (*PDHA1*): c.479T>G (p.Phe160Cys) in the patient. The variant c.479T>G caused a single nucleotide substitution on exon 5 and was predicted to be a disease-causing variant in the *in silico* analyses. We present the first report with a genetic analysis of a Vietnamese patient with pyruvate dehydrogenase E1-alpha deficiency (PDHAD). Sanger sequencing demonstrated that the patient inherited the variant from his mother who harbored the variant in a heterozygous state, but no PDHAD symptoms were observed in her. In addition, a prenatal test of the patient's mother revealed a fetus with a normal genotype. Furthermore, the patient's father and sister both carried a normal allele. Based on the American College of Medical Genetics criteria, the variant c.479T>G was predicted to be a likely pathogenic variant. Using the combination of the patient’s genotype and phenotype, he was definitively diagnosed with pyruvate dehydrogenase E1-alpha deficiency. Our findings expand the mutational spectrum of neurological disorders and provide the scientific basis for genetic counseling for the patient's family.

## Introduction

The pyruvate dehydrogenase complex (PDC) converts pyruvate into acetyl coenzyme A (acetyl-CoA). The human pyruvate dehydrogenase complex comprises pyruvate dehydrogenase (E1), dihydrolipoamide acetyltransferase (E2), dihydrolipoamide dehydrogenase (E3), E3-binding protein (E3BP), pyruvate dehydrogenase kinase (PDK), and pyruvate dehydrogenase phosphatase (PDP) (1). The pyruvate dehydrogenase E1 enzyme catalyzes the decarboxylation of pyruvate. Two E1-alpha proteins, encoded by the *PDHA1* gene, and E1-beta proteins, encoded by the *PDHB* gene, form the E1 enzyme. A pyruvate dehydrogenase complex deficiency (PDCD) induces a reduction of adenosine triphosphate (ATP) production and energy insufficiency, engendering neurological problems. Moreover, excessive pyruvate is converted into lactic acid, causing lactic acidosis. A pyruvate dehydrogenase complex deficiency encompasses four neurological phenotype groups, including neonatal encephalopathy with lactic acidosis, non-progressive infantile encephalopathy, Leigh syndrome, and relapsing ataxia (2).

Pathogenic variants in the *PDHA1* gene cause pyruvate dehydrogenase E1-alpha deficiency (PDHAD; OMIM 31270) and are responsible for 76%–85% of primary pyruvate dehydrogenase complex deficiency cases (3). PDHAD is characterized by lactic acidosis, delayed development, and neurological involvement. Pathogenic variants in the *PDHA1* gene create an abnormal E1-alpha protein that cannot interact with E1-beta to form the E1 enzyme, decreasing pyruvate dehydrogenase complex activity. The *PDHA1* gene is located on Xp22.1-p22.2, consisting of 11 exons (4). In the ClinVar database, a total of 549 *PDHA1* variants have been reported; however, only 142 are likely pathogenic or pathogenic variants (https://www.ncbi.nlm.nih.gov/clinvar; accessed on 12 March 2024). The likely pathogenic or pathogenic variants comprise 85 (59.86%) single nucleotide variants, 34 (23.94%) insertion variants, and 23 (16.20%) deletion variants.

The mode of PDHAD's inheritance is X-linked dominant. Men and women are almost similarly affected; however, the clinical presentation differs between the sexes (5). Neonatal lactic acidosis predominates in men, whereas the chronic neurological form is more common in women. Heterozygous women have variable X-inactivation patterns in different tissues and thus may present with varying symptoms (6). The increased lethality in some men with severe mutations and the X-inactivation pattern in women accentuate the clinical variability of an E1-alpha deficiency and its similarity to a recessive disease (7). In this study, we present the case of a boy diagnosed with PDHAD caused by a hemizygous variant in the *PDHA1* gene and presenting with severe metabolic acidosis, elevated serum lactate levels, hyperalaninemia, lactic acidosis, and globus pallidus lesions.

## Case presentation

**Figure 1:**
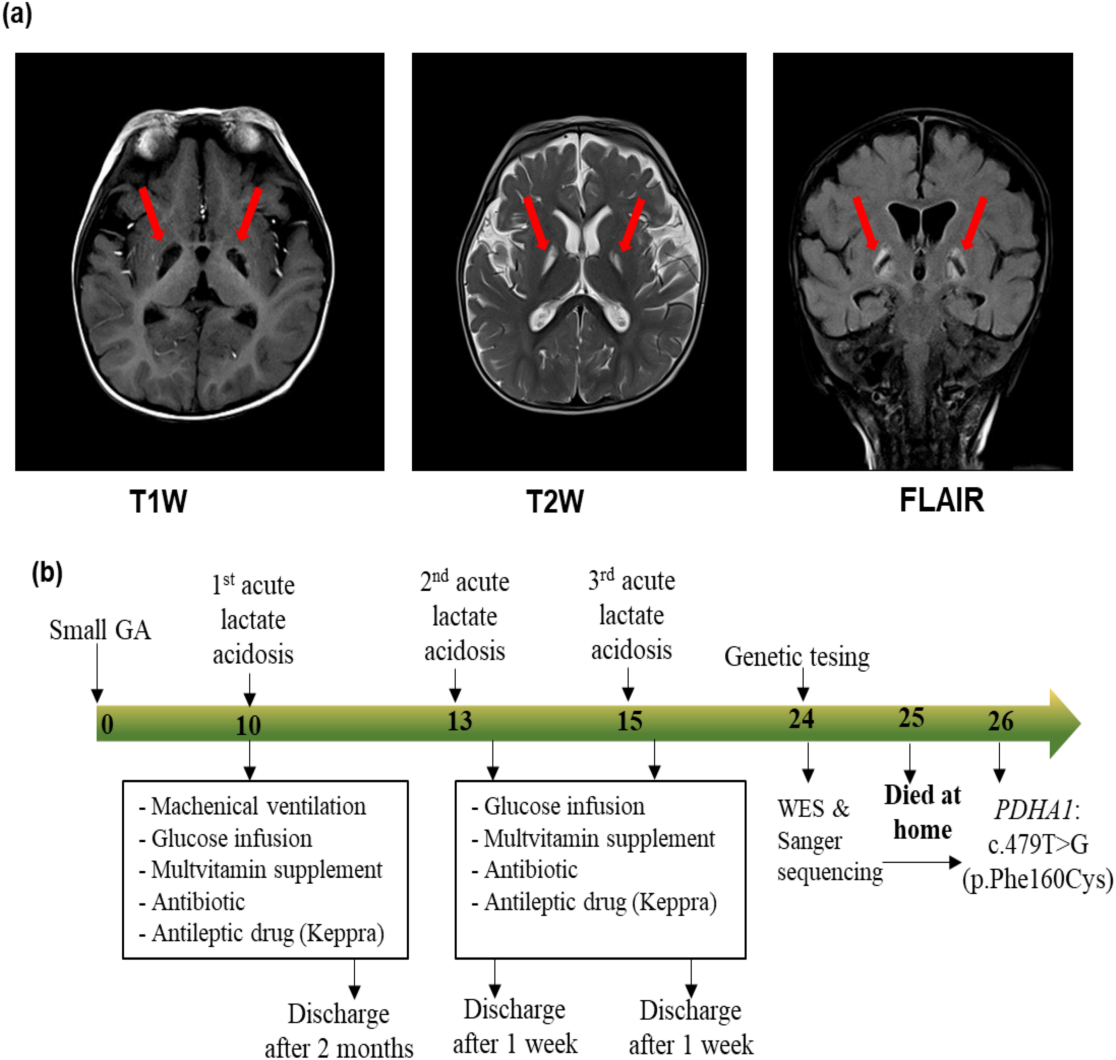
Magnetic resonance imaging of the brain **(a)** and timeline of medical events **(b)** of the proband. **(a)** Magnetic resonance imaging of the brain at 15 months of age showing globus pallidus lesions (hypointensity in the T1W image, and hyperintensity in the T2W image and FLAIR image). **(b)** The numbers in the arrow represent the months. The proband was small for his gestational age (GA), and presented with three episodes of acute lactate acidosis at 10, 13, and 15 months of age. At 24 months of age, whole blood was collected for genetic testing including whole-exome sequencing (WES) and Sanger sequencing. Two months later, a molecular diagnosis was achieved for the proband. However, at 25 months of age, the proband presented with tachypnea and coma and died at home.

The patient was the first child of healthy non-consanguineous Vietnamese parents. He was small for his gestational age because his birth weight was 2.2 kg at 38 weeks of gestation (Table 1). He was delivered by a cesarean section and had a normal Apgar score. He presented with global development delay with rolling at 7 months of age, hypotonia, and receptive language delay. At 10 months of age, he was admitted to the emergency department with lethargy, coma, hypotonia, and hyperventilation. His physical development was normal, with a weight of 9.0 kg (50th percentile) and a length of 73 cm (50th percentile). Three days before he was admitted to the hospital, he had ophthalmoplegia with no fever and no accompanying vomiting but was having feeding difficulties and lethargy. Based on the biochemical investigation at the admission, he had severe metabolic acidosis (pH: 6.99, HCO3^−^: 3 mmol/L, base excess (BE): −20 mmol/L), increased serum lactate (lactate: 5.8 mmol/L), normal blood glucose (5.6 mmol/L), normal blood ammoniac (30 μg/dl), normal transaminase (alanine aminotransferase (ALT): 38.5 UI/L; aspartate aminotransferase (AST): 12.7 UI/L), normal renal function (urea: 1.2 mmol/L; creatinine: 34.1 μmol/L), and an unremarkable complete blood count. Acylcarnitine and amino acid profiles revealed hyperalaninemia (661 μmol/L) and a low glutamic acid level (95.32 μmol/L). His urinary organic aciduria profile indicated lactic acidosis. Brain magnetic resonance imaging (MRI) showed two small lesions on the left cerebral peduncle and right quadrigeminal bodies: hypointensity on a T1-weighted (T1W) image, and hyperintensity on T2-weighted (T2W) and fluid-attenuated inversion recovery (FLAIR) images. Brain MRI at 15 months of age exhibited globus pallidus lesions (hypointensity on a T1W image and hyperintensity on the T2W and FLAIR images) (Figure 1a). He was suspected of having a mitochondrial disease. He was treated with a glucose infusion (5 mg/kg/min), correcting his acidosis. Furthermore, he was administered a multivitamin supplement, Keppra, and an antibiotic and placed on mechanical ventilation. He was discharged after 2 months. His condition at discharge time was described as follows: conscious, development delay, hypotonia, and no convulsions. His treatment continued with multivitamins and Keppra at home. He had two recurrent episodes of lactic metabolic acidosis at 13 and 15 months of age. For these episodes, he was administered a glucose infusion (5 mg/kg/min), multivitamin supplement, antibiotic, and antiepileptic drug (Table 1). He was discharged after receiving treatment for 1 week. Nevertheless, he developed an intellectual disability and dystonia. Genetic testing was performed when the patient was 24 months old. However, the patient died at home at 25 months of age before the genetic diagnosis was completed. Figure 1b summarizes the timeline of the patient's medical history.

**Table 1:** The clinical, biochemical, and molecular characteristics and the outcome of the patient as well as the interventions conducted.

Characteristics	Patient	Human phenotype ontology (HPO) term
Clinical and biochemical analyses		
Sex	Male	
History	Small for gestational age (Birth weight 2.2 kg at 38 weeks of gestation)	HP:0001518
	Global development delay (rolling at 7 months of age)	HP:001263
	Hypotonia	HP:0001252
	Receptive language delay and mental deterioration	HP:0010863
Age at admission	10 months	
Eyes	Ophthalmoplegia	HP:0000602
Musculature	Hypotonia	HP:0001252
Nervous system	Lethargy	HP:0001254
	Feeding difficulties	HP:0011968
	Hyperventilation	HP:0002883
Metabolism	Severe metabolic acidosis (pH: 6,99, HCO3^−^: 3 mmol/L, BE: −20 mmol/L)	HP:0001942
	Increased serum lactate (lactate: 5.8 mmol/L)	HP:0002151
	Hyperalaninemia (661 μmol/L)	HP:003348
	Low glutamic acid level (95.32 μmol/L)	
	Lactic acidosis	HP:0003128
Brain MRI at 10 months of age	Lesions on the left cerebral peduncle and right quadrigeminal bodies: hypointensity in T1W image, hyperintensity in T2W and FLAIR images	
Brain MRI at 15months of age	Globus pallidus lesions: hypointensity in T1W image, hyperintensity in T2W and FLAIR images	
Molecular analyses		
Gene	*PDHA1*	
GenBank transcript ID	NM000284.4	
Exon	5	
Position	chrX: 19371260	
cDNA change	c.479T>G	
Amino acid change	Phe160Cys	
Effect	Missense	
State	Hemizygous	
Inheritance	Maternal	
Database and literature	Not reported in the dbSNP, 1000 Genome Project, gnomAD, and in-house databases or in the literature	
SIFT	Deleterious with a score of 0	
Polyphen2	Probably damaging with a score of 1.000	
CADD	Deleterious with a Phred score of 31	
MutationTaster	Disease-causing with a probability of 0.999999	
ACMG classification	Likely pathogenic (PM2, PP1, PP3, and PP4)	
Interventions and outcome		
Events	Interventions	Outcome
First acute lactate acidosis at 10-month-old	– Mechanical ventilation – Acidosis correction by glucose infusion (5 mg/kg/min) – Multivitamin supplement (vitamin B1, L-carnitine, Coenzyme Q10, Biotin) – Antibiotic – Antiepileptic drug (Keppra)	– Patient recovered after 2 months of treatment. His condition at the discharge time: alert, development delay, hypotonia, and no convulsions
Second acute lactate acidosis at 13-month-old	– Glucose infusion (5 mg/kg/min) – Multivitamin supplement (vitamin B1, L-carnitine, Coenzyme Q10, Biotin) – Antibiotic – Keppra	– Patient was discharged after 1 week – Patient developed intellectual disability and dystonia
Third acute lactate acidosis at 15-month-old	– Glucose infusion (5 mg/kg/min) – Multivitamin supplement (vitamin B1, L-carnitine, Coenzyme Q10, Biotin) – Antibiotic – Keppra	– Patient was discharged after 1 week – Patient developed intellectual disability and dystonia
Home treatment	– Multivitamin supplement (vitamin B1, L-carnitine, Coenzyme Q10, Biotin) – Keppra	
At 25 months of age, he presented with tachypnea and coma and then died at home.		

Genomic DNA was isolated from peripheral blood according to the manufacturer's instructions. Whole-exome sequencing and bioinformatics analysis were conducted as described in a previous study (8). Screening for pathogenic variants involved the 1,135 genes associated with abnormal cerebral sub-cortex morphology. Variants with minor allele frequencies less than 0.01 in public databases [1000 Genomes Project or Exome Aggregation Consortium (ExAC)] were eliminated. Variants with a putative impact (in-frame deletions/insertions, frameshift, splice site, start/stop loss, stop-gained, or deleterious missense variants) were selected. Subsequently, the matches with the state of the variants in the patient (heterozygous or homozygous) and the inheritance of genes (autosomal dominant, autosomal recessive, or X-linked modes) were considered. The variants occurring in the in-house database were discarded. The potential disease-causing variants were assessed using the Mutation Taster and Combined Annotation Dependent Depletion (CADD) tools. In total, 5,508 variants were found in the 1,135 genes in the proband, of which 580 variants had a minor allele frequency of less than 0.01. Of the total number of variants, 25 variants with a putative impact (including 14 damaging missense variants, 5 in-frame deletions/insertions, 4 frameshift variants, 1 splice site variant, and 1 start loss variant) were analyzed further. Seven single heterozygous variants in the recessive genes and 11 variants appearing in the in-house database were removed. The pathogenicity of the seven remaining variants was predicted using *in silico* tools and only variant NM000284.4 (*PDHA1*): c.479T>G (p.Phe160Cys) was predicted as a “disease-causing” variant (Table 1). The variant is not documented in the dbSNP, 1000 Genomes Project, gnomAD, and in-house databases, or in the literature.

**Figure 2:**
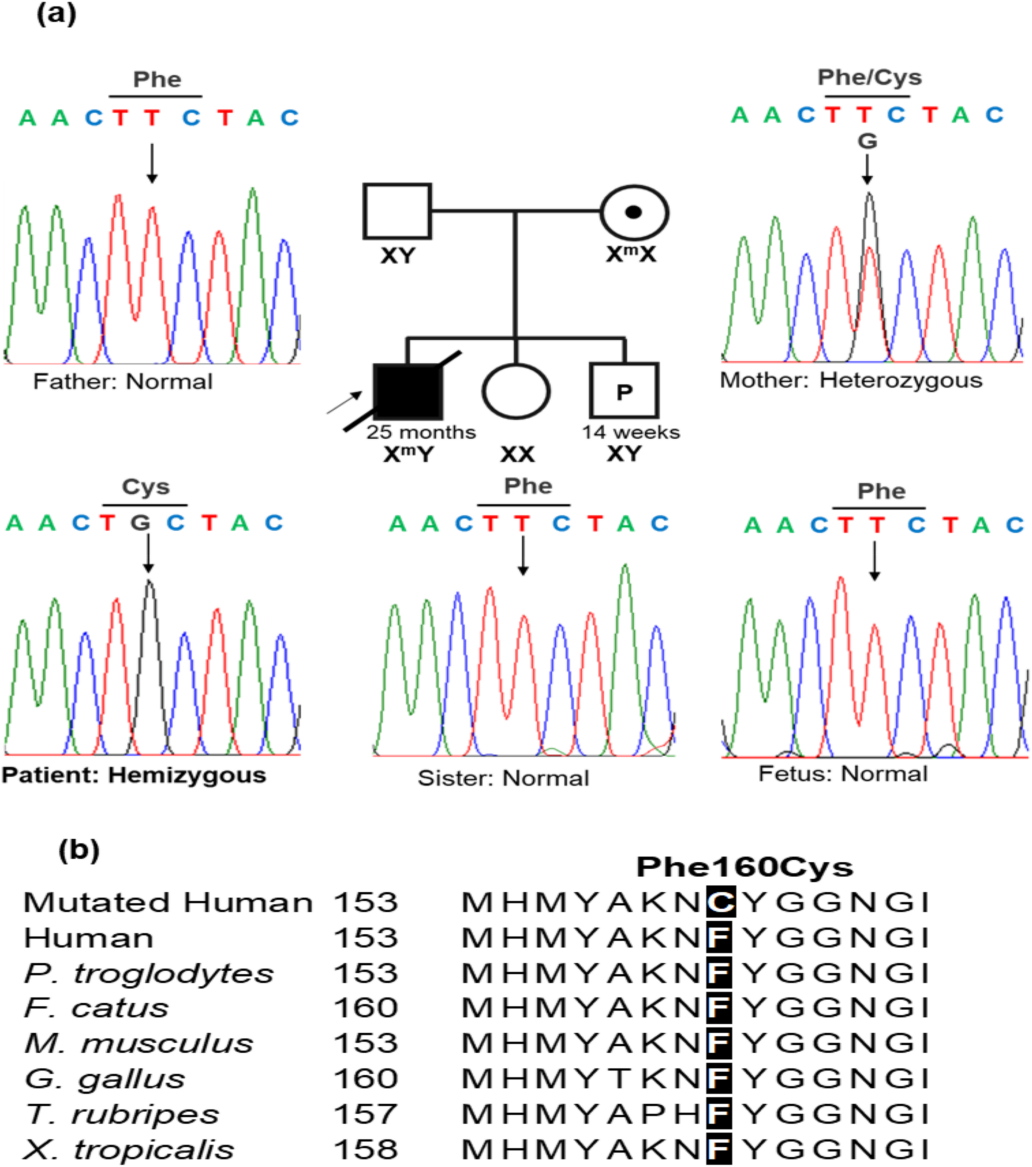
A *PDHA1* variant was confirmed by Sanger sequencing. **(a)** A DNA sequencing electropherogram in the patient's family, the variant NM000284.4 (*PDHA1*): c.479T > G (p.Phe160Cys) was hemizygous in the patient. The mother carried the variant in a heterozygous state, while the father, sister, and fetus harbored normal alleles. **(b)** The alignment of multiple PDHA1 amino acid sequences showed the highly conserved amino acid at the Phe160 position.

Sanger sequencing was conducted in the patient's family to identify the presence of the variant NM000284.4 (*PDHA1*): c.479T>G (p.Phe160Cys), which is located in exon 5 of the *PDHA1* gene. The primer pair for exon 5 amplification comprised a forward primer (5′-GACTGAACTGGCCTCTGTGT-3′) and a reverse primer (5′-AGTTGTCTGGGGCTGTGAAA-3′). The results demonstrated that the patient harbored the variant c.479T>G (p.Phe160Cys) in a hemizygous state (Figure 2a). The mother had the variant in a heterozygous state even though she did not exhibit any symptoms of PDHAD. The patient inherited the variant c.479T>G (p.Phe160Cys) from his mother, as his father and sister did carry the variant. The family received genetic counseling for X-linked dominant inheritance. The asymptomatic mother harbors one mutant allele and the father has normal alleles. Each of the couple's offspring will inherit one X-chromosome from the mother and may inherit the variant *PDHA1*: c.479T>G (p.Phe160Cys) with a 50% chance of being affected. Therefore, when the patient's mother was pregnant, she consented to prenatal testing. The patient's mother was screened prenatally through amniocentesis under ultrasound guidance at 14 weeks of gestation, with 15 ml amniotic fluid acquired from the abdominal wall. Of this, 10 ml was centrifuged to collect cells for culturing in AmnioMax C-100 Complete Medium (Thermo Fisher, USA). DNA was extracted from the cells after 14 days of culture using the QIAamp DNA Blood Mini Kit (Qiagen, Germany). Sanger sequencing was then performed to determine the presence of the disease-causing variant in the fetus. The fetus exhibited a normal genotype and the pregnancy continued (Figure 2a). The c.479T>G (p.Phe160Cys) variant was classified as a likely pathogenic variant using the American College of Medical Genetics (ACMG) criteria for assessing pathogenicity (9) because it meets one moderate (PM2) and three supporting rules (PP1, PP3, PP4).

## Discussion

PDHAD exhibits allelic heterogeneity, with at least 100 mutations, primarily including missense, small in-frame indel, and frameshift variants, which have been identified by sequencing the entire PDHA1 coding region. Missense variants are more common primarily found in exons 3–9 (6, 10, 11). The defective enzymes are expressed in all the cells in hemizygous males, and the residual PDC activity depends on the mutation's effect. Typically, nonsense variants may induce partially active enzyme production. Conversely, the defective enzymes are only found in some cells of heterozygous women who can tolerate more severe mutations and experience more frequent frameshift mutations than men. Heterozygous women with nonsense mutations are usually asymptomatic or have a milder clinical phenotype and their life expectancy could be 20–30 years (5). However, some heterozygous cases were severely impacted by nonsense mutations, indicating the differential impact of each mutation on the structure and function of E1α (11, 12).

Recently, several studies have identified pyruvate dehydrogenase complex deficiency concurrently with multiple lesions on MRI during different stages of brain development (13) or with a combination of alanine/leucine l ≥4.0 and proline/leucine ≥3.0 from dried blood spot specimens (14). Nevertheless, distinguishing a PDCD based on clinical and biochemical features is challenging. Whole-exome sequencing is a suitable method for definitively diagnosing a PDCD (15).

Our patient presented with progressing globus pallidus lesions in brain MRI. The biochemical investigation demonstrated severe metabolic acidosis, increased serum lactate, hyperalaninemia, and lactic acidosis. Based on these clinical characteristics, the patient was initially diagnosed with mitochondrial disease. However, WES identified a likely pathogenic variant, c.479T > G (p.Phe160Cys), in the patient's *PDHA1* gene. The molecular analyses supported a definitive diagnosis of PDHAD for the patient. However, the molecular diagnosis was completed after the patient died. Therefore, he could not receive additive treatment such as thiamine.

Variants in *PDHA1* exhibit variable expressivity. The severity of phenotypic presentation can vary based on the residual enzymatic activity caused by the *PDHA1* variant (10). The variant c.479T>G (p.Phe160Cys) converted phenylalanine to cysteine at the position of amino acid 160, a highly conserved one (Figure 2b). Therefore, the variant c.479T>G (p.Phe160Cys) may affect the function of the protein. The c.479T>G (p.Phe160Cys) variant is located in exon 5 with 10 likely pathogenic/pathogenic single nucleotide substitutes, including NM_000284.4 (*PDHA1*): c.422G>A (p.Arg141Gln), c.455C>T (p.Ser152Leu), c.461A>G (p.His154Arg), c.465G>T (p.Met155Ile), c.482A>G (p.Tyr161Cys), c.483C>T (p.Tyr161Tyr), c.491A>G (p.Asn164Ser), c.498C>T (p.Ile166Ile), c.499G>A (p.Val167Met), and c.506C>T (p.Ala169Val). The c.482A>G (p.Tyr161Cys) and c.483C>T (p.Tyr161Tyr) variants have been observed in three patients (16, 17). In the vicinity of the non-consensus splice sites, synonymous mutations within exons may activate aberrant splicing by disrupting exonic splicing enhancer motifs (16, 17). Our patient and the three reported patients had variable phenotypes in brain MRI, muscle tone, neurodevelopment, and disease severity (Table 2). Our patient and the boy harboring the c.483C>T (p.Tyr161Tyr) variant died at 25 months and 16 days of age, respectively, whereas the other two patients lived to at least 5 years of age (Table 2). The literature indicates that the c.506C>T variant has been found in three individuals with presentations aligning with *PDHA1*-related disease and it can be categorized as a pathogenic variant for *PDHA1*-related pyruvate dehydrogenase deficiency in an X-linked manner (18). Furthermore, previous functional studies have demonstrated the damaging effect of the c.498C>T variant regarding the incomplete inclusion of *PDHA1* exon 5 (17). To our knowledge, our study is the first to describe a *PDHA1* variant in a Vietnamese family. However, further research must verify the relationship between c.479T>G (p.Phe160Cys) and PDHAD.

**Table 2:** Phenotype of four patients with changes in residues 160 and 161 of PDHA1.

	Patient 1	Patient 2	Patient 3	Patient 4
Sex	Male	Female	Male	Male
Variant	c.479T>G (p.Phe160Cys)	c.482A>G (p.Tyr161Cys)	c.483C>T (p.Tyr161Tyr)	c.483C>T (p.Tyr161 Tyr)
Reference	This study	(16)	(17)	(17)
Presentation	– Delayed development – Hypotonia	– Delayed motor and cognitive development – Spastic cerebral palsy – Epileptic seizure – Increased muscle tone in the lower extremities	– Abnormal movements – Bradycardia – Hypotonia	– Failure to thrive – Normal cognitive development – Hypotonia – Mild spasticity persisted – Seizure
Brain MRI	Globus pallidus lesions at 15 months of age	High signal intensity lesions in both basal ganglia suggestive of Leigh disease at 4 years of age	Asymmetrical parieto-occipital T2 hypersignals, associated with meningeal hemorrhages	–
Biochemical test	Hyperlactatemia, hyperalaninemia	Hyperlactatemia, hyperalaninemia, elevated pyruvic acid	Hyperlactatemia, hyperalaninemia, hyperlactatorrachia, and hyperlactaturia	Hyperlactatemia.
Recurrence	Recurrent severe metabolic acidosis	Recurrent epileptic seizures.	Recurrent severe metabolic acidosis	Recurrent seizures
Life expectancy	Died at 25 months of age	>5 years of age	Died at 16 days of age	>5 years of age

Our study demonstrated that the mother harbored the c.479T>G (p.Phe160Cys) variant in a heterozygous state. Even though none of the PDHAD symptoms were observed, the patient died at home at 25 months of age due to symptoms of acute metabolic acidosis, including tachypnea and coma. This patient's death can be attributed to the X-chromosome inactivation pattern in the mother, where the mutant allele is inactivated (5). In addition, genetic counseling was given to the couple. Because PDHAD is an X-linked dominant disorder (5), the couple's risk of having affected offspring is 50%. The affected male offspring would present with more severe lactic acidosis compared with female offspring. Therefore, genetic testing should be performed on the couple's new offspring to determine the presence of a mutant allele for appropriate genetic counseling and early medical intervention. During the mother's pregnancy, prenatal testing was performed. The result indicated that the fetus did not carry the variant. Such information helped the mother decide to continue her pregnancy. The couple also received preconception genetic counseling and were informed of the anticipated burden resulting from an affected child. In addition, the mother did not exhibit any symptoms of PDHAD. However, she is at high risk of late-onset PDHAD. The manifestations may include migraine, paralysis (19), epilepsy, polyneuropathy, and muscle weakness (20). Thus, such manifestations must be monitored for when the mother suffers any infection.

Mitochondrial disorders present with an extensive clinical spectrum, with heterogeneity in biochemical and genetic defects (21). Even though the clinical symptoms seem identical, mutations in different mitochondrial or nuclear genes may be the cause. Nonetheless, the same mutation can induce different phenotypes, complicating the diagnosis of mitochondrial disorders. Conversely, the same mutation may cause different phenotypes, complicating the diagnostic process (22). WES is the most advanced next-generation sequencing technique for identifying genetic defects in mitochondrial disorders (22). In our study, we described how to use WES in routine diagnostics. Our results show that WES technology can be successfully implemented as the most advanced molecular diagnostic test for mitochondrial disorders and neurological problems. Moreover, our findings reveal that clinical and biochemical phenotyping analysis is pivotal for successfully using WES to diagnose each patient.

Our study presents the first reported case of a Vietnamese boy with the novel c.479T>G (p.Phe160Cys) variant in the *PDHA1* gene. Furthermore, our study expands the mutation spectrum of the *PDHA1* gene for inducing pyruvate dehydrogenase E1-alpha deficiency and strengthens the role of whole-exome sequencing for molecular investigations in neurological problems.

## Data Availability

The datasets presented in this study can be found in online repositories. The names of the repository/repositories and accession number(s) can be found in the article/Supplementary Material.
